# The Marine Fungi *Rhodotorula* sp. (Strain CNYC4007) as a Potential Feed Source for Fish Larvae Nutrition

**DOI:** 10.3390/md15120369

**Published:** 2017-12-01

**Authors:** M. Barra, A. Llanos-Rivera, F. Cruzat, N. Pino-Maureira, R. R. González-Saldía

**Affiliations:** 1 Marine Biotechnology Unit, Department of Oceanography, Faculty of Natural and Oceanographic Sciences, Universidad de Concepción, Casilla 160-C, 4030000 Concepción, Chile; mariajosbarra@udec.cl (M.B.); alllanos@udec.cl (A.L.-R.); fecruzat@udec.cl (F.C.); napino@udec.cl (N.P.-M.); 2 Center for Oceanographic Research COPAS Sur-Austral, Universidad de Concepción, Casilla 160-C, 4030000 Concepción, Chile; 3 Doctoral Program in Aquatic Living Resources (MaReA), Faculty of Natural and Oceanographic Sciences, University of Concepción, Casilla 160-C, 4030000 Concepción, Chile

**Keywords:** *Rhodotorula*, DHA, EPA, *Danio rerio*, larvae nutrition, RNA/DNA ratio

## Abstract

Fish oil is used in the production of feed for cultured fish owing to its high polyunsaturated fatty acid content (PUFA). The over-exploitation of fisheries and events like “El Niño” are reducing the fish oil supply. Some marine microorganisms are considered potentially as alternative fatty acid sources. This study assesses a strain of *Rhodotorula* sp. (strain CNYC4007; 27% docosahexaenoic acid (DHA) of total fatty acids), as feed for fish larvae. The total length and ribonucleic acid (RNA)/deoxyribonucleic acid (DNA) ratio of *Danio rerio* larvae was determined at first feeding at six and 12 days old (post-yolk absorption larvae). Larvae fed with microencapsulated *Rhodotorula* sp. CNYC4007 had a significantly higher RNA/DNA ratio than control group (C1). At six days post-yolk absorption group, the RNA/DNA ratio of larvae fed with *Rhodotorula* sp. bioencapsulated in *Brachionus* sp. was significantly higher than control group fed with a commercial diet high in DHA (C2-DHA). Finally, at 12 days post-yolk absorption, the RNA/DNA ratio was significantly higher in larvae fed with *Rhodotorula* sp. CNYC4007 and C2-DHA (both bioencapsulated in *Artemia* sp. nauplii) than in control group (C1). These results suggest that *Rhodotorula* sp. CNYC4007 can be an alternative source of DHA for feeding fish at larval stage, providing a sustainable source of fatty acids.

## 1. Introduction

Marine resources for fishmeal and fish oil have been exploited beyond maximum sustainable yields [1], and the rate of exploitation is increasing by 8.8% per year [2]. One of the fish oil uses is the production of feed for aquaculture [3], an area that has expanded significantly, resulting in the rising demand for fish oil, which has consequently increased the price of this product [4]. Fish oil is used because of its high nutritional value and essential polyunsaturated fatty acid content (PUFA), among which are docosahexaenoic acid (C22:6, DHA), eicosapentaenoic acid (C20:5, EPA) and docosapentaenoic acid (C22:5, DPA), [5]. Freshwater fish generally have sufficient elongase and desaturase activities to produce these fatty acids from the 18C precursor. On the contrary, marine fish have a very limited capacity to synthesize these fatty acids [6], hence a strict requirement for long-chain PUFA, eicosapentaenoic, docosahexaenoic and arachidonic acids (essential fatty acid; EFA). Therefore, when these fatty acids are not synthesized in fish they should be incorporated into the diets of the larval-to-adult stages of the species that are of commercial interest [7,8]. Their presence is important for normal growth, reproduction, metabolic functions, feeding efficiency and immunocompetence [9]. Specifically, DHA has a high biological value during larval development and is selectively incorporated in neural tissue, contributing to pigmentation and visual acuity [10]. Lipids are maternally supplied and sustain both embryonic and yolk sac larvae development, but when the yolk absorption is completed and first feeding is started an exogenous source of PUFA is required [3]. Then, at the time of first feeding the larvae should receive a diet that covers all the nutritional requirements, so for aquaculture species the research focus has been to improve the nutritional content of artificial diets for larvae [7,11], as diets lacking in nutritional content result in low survival rates [12]. 

Fatty acids have also been encapsulated in rotifers (*Brachionus* sp.) and *Artemia* sp. nauplii [13,14], these organisms allow for the passing of essential nutrients to the larvae [15,16]. Morphological and histological parameters, as well as molecular ratios like the ribonucleic acid (RNA)/deoxyribonucleic acid (DNA) ratio, have been used to determine if effectively delivered nutrients improve the nutritional condition of the target species [17]. Theoretically, RNA levels in cells vary in relation to protein synthesis [18], while DNA concentrations remain constant even during starvation [19]. Therefore, the RNA/DNA ratio is a potential indicator of cellular protein synthesis and growth [17].

The declining supply of traditional sources of essential fatty acids and their high cost has led to the search for alternative sources [20], among which are vegetable seed oils [21] that contain alpha-linolenic acid (ALA, 18:3 ω-3), a precursor of DHA and EPA that is assimilated, but not necessarily transformed, into fatty acids [22]. However, it has not been fully possible to enhance these oils for their use in aquaculture as anti-nutritional components have been found in them that affect lipid homeostasis and energy metabolism [23], along with weakening immune response [24]. Another alternative to fish oil is single-cell oils (SCO) from one-cell organisms like bacteria, algae, fungoid protists and marine fungi, which have great potential because of their high essential long-chain unsaturated fatty acid content [25,26]. Marine fungi has also been described as producers of carotenoids, omega-3 fatty acids, including DHA and EPA [27], and omega-6 acids like arachidonic acid (20:04 ω6, AA) [28].

Given their characteristics, strains of marine microorganisms constitute as alternative sources that can partially or totally replace fish oil for the nutrition of fish [25,29,30]. For example, *Schizochytrium* sp., a strain that produces a high level of DHA (28% of its dry weight), has been highly studied because it has been proven with juvenile *Salmo salar* that the complete replacement of fish oil with this strain does not negatively affect growth, immune response, and digestibility of nutrients [25]. Its use with other marine species under intensive cultivation like *Sparus aurata* [31] has also been tested and with positive results. *Schizochytrium* sp. has therefore been proposed as a sustainable source of fatty acids [25].

A strain of *Rhodotorula* sp. (CNYC4007) from the Humboldt Current System off Central Chile was isolated and grown in a bioreactor. The strain, which has high levels of DHA, EPA and carotenoids [27], could be considered as a suitable source of omega-3 for the aquaculture demand. In reference to the above the aim of this study was to evaluate the use of strain CNYC4007 for feeding *Danio rerio* larvae. The zebrafish (*D. rerio*) has been widely studied in various fields of biology [32], and in recent years has been proposed as a possible model in studies of nutrition and growth in fish [33], especially as a model in aquaculture research [34]. However, despite potential limitations due to species-specific differences in fatty acid metabolism, zebrafish is a useful tool in the initial screening of new supplies for larval nutrition with lower cost and rearing times than the use of aquaculture species [35]. To date, research has been provided on the anti-nutritional aspects of some alternative ingredients [36] and individualized the requirements for mineral and trace element requirements that have been established for adequate larval development and growth in zebrafish that could benefit other fish species [16]. Therefore, we used zebrafish larvae as a model in the first step to determine the potential use of *Rhodotorula* sp. (CNYC4007) in larval nutrition according to the usual feeding protocols implemented in larval rearing.

## 2. Results

### 2.1. RNA/DNA Ratio as a Proxy for the Nutritional Condition of Danio rerio

**Figure 1:**
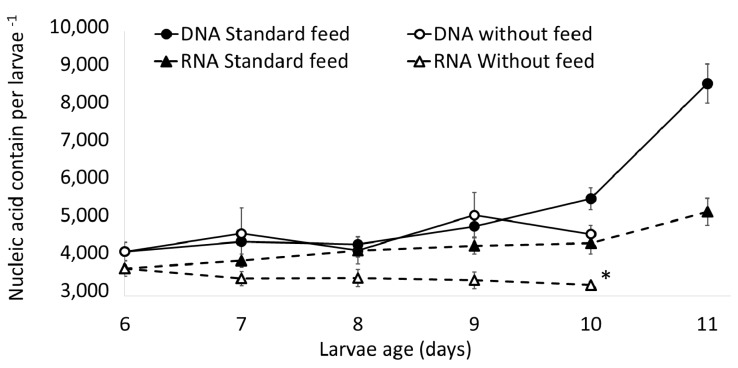
Average nucleic acid content (ng) versus larvae age of *Danio rerio* fed with standard feed compared to treatment with larvae in starvation. Each point represents the average of four larvae per treatment. * *p* < 0.05.

**Figure 2:**
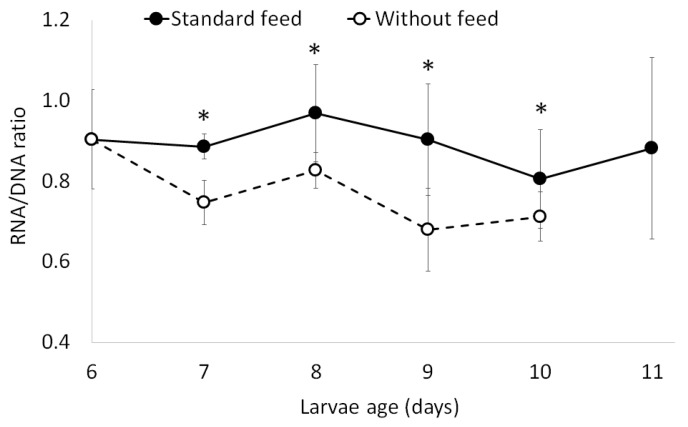
Ribonucleic acid (RNA)/deoxyribonucleic acid (DNA) ratio versus test of larvae of *Danio rerio* fed with standard feed compared to that of larvae in starvation. The daily average is based on four larvae per treatment. * *p* < 0.05.

The total DNA content per individual did not present a significant difference among live larvae up to the 5th day five of the experiment, when the larvae were 10 days old (Kruskal-Wallis test, *p* = 0.0565). As well, no significant differences were observed for the same parameter among the treatments during the days of the assay (Figure 1). However, there was 100% mortality rate by 6th day of the larvae in starvation (11 days old), while there was 100% survival rate among the larvae that were fed. The quantity of RNA in larval tissue (Figure 1) progressively increased over the course of the treatment with the standard feed, presenting significant differences throughout the assay (one-way analysis of variance (ANOVA), F-statistic = 31.09, *p*-value = 4.18 × 10^−5^). In contrast, RNA content in the tissue of the larvae under the starvation treatment decreased over the course of the assay. Comparing RNA content when the larvae were 10 days old (Figure 1), a significant difference between larvae fed with the standard feed (C1) and those in starvation (one-way analysis of variance (ANOVA), F-statistic = 11.6, *p*-value = 0.00362) was found. The average of the RNA/DNA ratio of the treatment with the standard feed (Figure 2) when larvae were 10 days old (5th day of the treatment) was 0.81 ± 0.12, and the starvation treatment had a lower average (0.71 ± 0.06). This ratio presents significant differences between the treatments (two-way ANOVA, F-statistic = 5.873, *p*-value = 0.0210).

### 2.2. Assay with Six-Day-Old Larvae (First-Feeding Larvae, One Day Post-Yolk Absorption)

**Figure 3:**
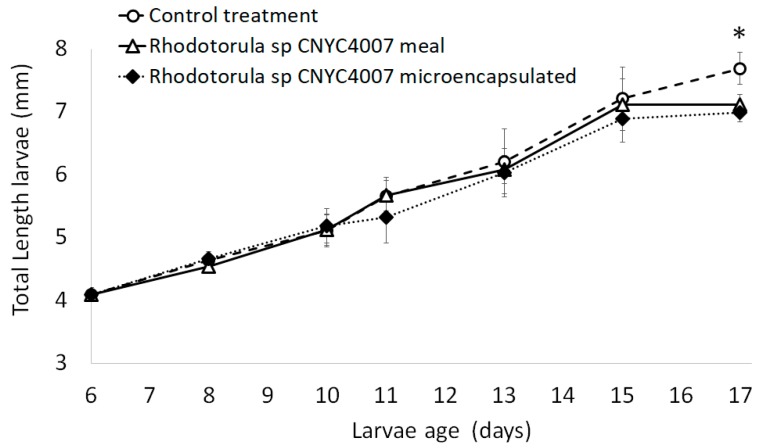
Total length versus larvae age of *Danio rerio* at first feeding larvae. The daily average is based on six larvae per treatment. * *p* < 0.05.

**Figure 4:**
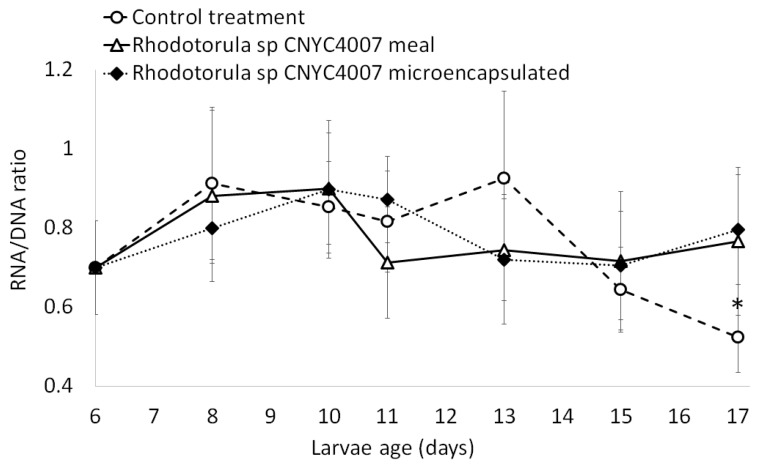
RNA/DNA ratio versus larvae age of *Danio rerio* at first feeding larvae. The daily average daily is based on six larvae per treatment. * *p* < 0.05.

There were significant differences among the three treatments (one-way ANOVA, F-statistic = 1809, *p*-value = 2 × 10^−16^) by the final day of the assay (Figure 3). A significantly greater average larval length (7.7 ± 0.3 mm) was obtained with the control feed (control group) than with the microencapsulated *Rhodotorula* sp. CNYC4007 and with the flour lyophilized of *Rhodotorula* sp. CNYC4007 (7.0 ± 0.2 mm and 7.1 ± 0.2 mm, respectively; Tukey test; *p*-value = 0.000125 for both treatments). There were no significant differences in the total length between larvae fed on flour and those fed on microencapsulated *Rhodotorula* sp. CNYC4007 (Tukey test, *p* = 0.3641). With respect to the molecular analysis, the three treatments did not present any significant differences over the course of the assay (Kruskal-Wallis *p* = 0.148), except for the final day (Figure 4) when the RNA/DNA ratios in the larvae in both the treatments with microencapsulated *Rhodotorula* sp. CNYC4007 and with lyophilized flour (which had the same ratios; 0.8 ± 0.14) were higher than that of the larvae in the control group (0.53 ± 0.09). 

### 2.3. Feeding Six-Day-Old Larvae (Post-Yolk Absorption) with Encapsulation in Brachionus *sp.*

There were no significant differences in the total length between the larvae (Table 1) fed with rotifers without enrichment (control treatment), rotifers enriched with C2-DHA (positive control) and rotifers enriched with *Rhodotorula* sp. CNYC4007 (one-way ANOVA, F-statistic = 0.89, *p*-value = 0.41), nor were significant differences observed in the lengths of larvae from the different treatments on the final day of the assay (one-way ANOVA, F-statistic = 0.34, *p*-value = 0.72). Similarly, there were no significant differences in the RNA/DNA ratios of the larvae from the three treatments (Kruskal-Wallis *p* = 0.148), except for 7th day of the assay when larvae are 12 days old (Table 2), the RNA/DNA ratios of larvae fed with *Brachionus* sp. (control treatments) and *Brachionus* sp. enriched with C2-DHA (positive control) were significantly lower than those of larvae fed with *Rhodotorula* sp. CNYC4007 (Tukey test, *p* = 0.0017 and *p* = 0.0087, respectively). There were no significant differences (Tukey test, *p* = 0.6803) between the treatments with *Brachionus* sp. (control treatment) and *Brachionus* sp. enriched with C2-DHA (Table 2).

### 2.4. Experiments with 12-Day-Old Larvae

There were no significant differences in the total length of these larvae (Table 1) used in the treatment with the enriched *Artemia* sp. nauplii (one-way ANOVA, F-statistic = 0.713, *p*-value = 0.493), either throughout or on the final day of the assay (one-way ANOVA, F-statistic = 0.869, *p*-value = 0.43). However, by the final day there were significant differences in the RNA/DNA ratio (Table 2) between the control group and the group treatment with nauplii enriched with C2-DHA and with the strain CNYC4007 (Tukey test, *p*-value = 0.0003 and *p*-value = 0.005, respectively). Finally, there were no significant differences on the final day of assay between the treatment with *Artemia* sp. nauplii enriched with C2-DHA and that with nauplii enriched with CNYC4007 (Tukey test, *p*-value = 0.39).

### 2.5. DHA and EPA Enrichment of Artemia *sp.* nauplii

**Figure 5:**
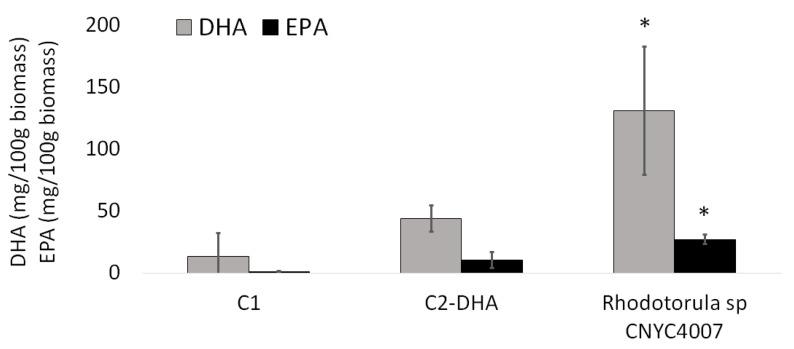
Average DHA and eicosapentaenoic acid (EPA) concentrations (± S.D.) in *Artemia* sp. nauplii without enrichment (C1), fed feed rich in DHA (C2-DHA) and fed with the strain *Rhodotorula* sp. CNYC4007. * *p*-value < 0.05.

There was a significant high DHA concentration (Figure 5) in nauplii enriched with the strain CNYC4007 (*Rhodotorula* sp.) compared to those without enrichment or enriched with C2-DHA (one-way ANOVA, *p*-value = 0.0036). There were no significant differences in DHA concentrations (*p*-value > 0.05) between nauplii without enrichment (control treatment) and those fed with C2-DHA. The same was observed with EPA concentrations, which were significantly high for nauplii fed with the strain CNYC4007 (*Rhodotorula* sp.) compared to those of nauplii without enrichment or enriched with C2-DHA (one-way ANOVA, *p*-value = 0.00017). There were no significant differences in EPA concentrations (*p*-value > 0.05) between *Artemia* sp. nauplii without enrichment (control treatment) and those fed with C2-DHA.

## 3. Discussion

The use of strains of marine microorganisms in commercial fish feed and other applications, such as a source of food for human consumption, requires in vivo validation. The first step is to rule out toxic effects and then verify the nutritional contribution to the species under study. The condition index of Fulton [37] is generally used for organisms that are sufficiently large to be easily weighed and measured. However, with fish larvae the determination of difference in a very small body mass tends to lack precision. The present work studied the effect of the strain CNYC4007 of the marine basidiomycete *Rhodotorula* sp. [27] in the RNA/DNA ratio, which was used as a proxy for the nutritional state of *Danio rerio* larvae. The larvae were fed in their different stages of development and the strain was administered in three forms (flour, microencapsulated, and bioencapsulated).

The results show the efficacy of the RNA/DNA method to evaluate the nutritional condition of larvae in the experiments carried out, given that it effectively distinguished between the conditions of the larvae that were fed and those in starvation (Figure 1 and Figure 2). This method is relevant and has been used above all with animals at the size scale of zooplankton [22,38] and fish larvae [17,39,40]. This ratio is considered a metabolic index and has been used as a measurement of individual growth rate and nutritional state [18,41]. The increase in RNA concentrations in larvae fed with standard feed (Figure 1) suggests there was protein synthesis and with this a progressive increase in larval size throughout the assay, as has been observed with other species like *Sardina pilchardus*, *Engraulis encrasicolus*, *Atherina presbyter* and *Paralichthys orbignyanus* in different development stages [42]. This pattern concurs with that described by Chung and Segnini [43], who found that RNA levels were higher in rainbow trout larvae that were fed, as opposed to those in starvation. In the present work, average DNA levels per individual were observed to be constant during technical standardization assays (Figure 1). It has been described that DNA levels do not vary under stress, whether caused by starvation or environmental conditions [44]. Coincident with our results, Ben Khemis et al. [17] proposed that RNA/DNA is a useful molecular tool to evaluate larval condition in aquaculture. 

In all of the assays the RNA/DNA ratio in the larval stages of *Danio rerio* ranged between 0.7 and 2. These values were similar to those obtained in *Oncorhynchus mykiss* (1.5–2.5) [45]. It was observed that the ratio was sensitive to changes in the diet composition; this concurs with what has been described in rainbow trout, where the RNA/DNA ratio of larvae varied in response to distinct protein compositions included in their diet (40–50%) [45]. There were no differences among the feeding treatments when only considering larval length, although there were differences in nutritional state.

The first feeding assay with *Danio rerio* larvae indicates that the strain *Rhodotorula* sp. CNYC4007 can be used effectively for this purpose. Larvae fed with flour and microcapsules had similar lengths (Figure 3) and were in a similar nutritional condition (Figure 4), indicating that the two forms of administrating feed can be considered as appropriate. The microencapsulated feed contained stabilizers (maltodextrine and capsule) that protected the fatty acids against moisture, light and other environmental factors [46], which because of storage or contact with water can deteriorate the feed [47]. Furthermore, the preparation of microcapsules only required 4% of the microorganisms biomass required for the flour lyophilized with *Rhodotorula* sp. CNYC4007 and its physical properties allowed the feed to remain longer on the surface of the water, which facilitates feeding the larvae, in contrast to the flour, which decants rapidly. Thus, the microencapsulation method to administer *Rhodotorula* sp. (CNYC4007) is recommended for first-feeding the larvae because it is more stable, offers better protection of fatty acids and is more bioavailable than the lyophilized flour method (100% of flour), the latter thus requiring a large quantity of microorganisms to achieve the same effect.

As the larvae grow their mouth size and nutritional requirements increase, because of which the quantity and nutritional composition of the feed must be modified [48]. Because of this, in aquaculture, especially with marine species, live prey such as rotifers and *Artemia* sp. nauplii are used. However, these have low levels of polyunsaturated fatty acids. Rotifers have 4.19% arachidonic acid and 2.29% docosapentaenoic acid of total fatty acids, with the absence of DHA [16], and *Artemia* sp. nauplii have low levels of EPA with the absence of DHA [47]. Consequently, it is necessary to enrich these organisms for use as larval feed [13]. With larvae at six days post-yolk absorption (six days old), *Rhodotorula* sp. CNYC4007 was bioencapsulated in rotifers. The results indicate that the strain is not toxic for *Brachionus* sp., *Artemia* sp. nauplii or zebrafish larvae (0% mortality in the assay). In the case of larvae, its presence in the digestive tract was confirmed and larvae presented a better molecular condition than larvae fed on rotifers without enrichment. This concurs with the results obtained with bioencapsulation in rotifers of *Schizochytrium mangrovei*, a marine fungoid used to enrich live prey [14]. By comparing the fatty acid content of rotifers with and without enrichment, the authors observed that the rotifers absorbed significant levels of the DHA present in the lyophilized of *S. mangrovei* [14]. Barclay and Zeller [49] also found the DHA content in rotifers fed with *Schizochytrium* sp. was significantly higher than that of control rotifers fed with beer yeast. *Schizochytrium* sp. is one of the ingredients included in the formulation of feed, termed in this study as C2-DHA, which was considered as a positive control in experiments in the present work. Both *Schizochytrium mangrovei* [14] and the strain *Rhodotorula* sp. CNYC4007, used in this experiment, have high percentages of DHA (31.53% and 27%, respectively, of total fatty acids).

The incorporation of the bioencapsulated strain CNYC4007 in feed resulted in significantly higher concentrations of polyunsaturated fatty acids in *Artemia* sp. nauplii than in nauplii fed with bioencapsulated C2-DHA (Figure 5). Consequently, *Rhodotorula* sp. CNYC407, as well as *Schizochytrium* sp. [50], represent alternative species for enriching nauplii, even though the percentages of DHA and EPA as part of the total of fatty acids differ between the two species (27% and 43% of DHA; 7.2% and 2.8% of EPA for *Rhodotorula* sp. and *Schizochytrium* sp., respectively). With larvae at 12 days post-yolk absorption, the RNA/DNA ratio of larvae in the control treatment was lower than that of larvae in the treatment with *Artemia* sp. nauplii enriched with feed rich in DHA (C2-DHA) and with the strain *Rhodotorula* sp. CNYC4007. The bioencapsulation of highly unsaturated fatty acids of the omega-3 series in *Artemia* sp. nauplii has been shown to improve growth and survival of marine fish larvae [51], which concurs with what was observed with the enrichment of *Artemia* sp. with the strain studied in this work. 

Essential fatty acid requirements vary qualitatively and quantitatively with environmental origin and during the ontogeny of fish, with early developmental stages and broodstock being critical periods [3]. Specifically, as there is evidence that n-3 HUFA (highly unsaturated fatty acids) and DHA may be more important and, possibly, essential in the larvae of some species of freshwater fish compared with adults. In marine species, larvae are characterized by having a greater requirement for n-3 HUFA than juvenile and pre-adult fish, although there are relatively few species where the requirements at larval and juvenile stages can be directly compared. Notwithstanding the foregoing, scarce data exists in relation to EFA requirements (expressed as % dry diet), in larvae and early juvenile fish, with values that ranged between 1 to 5% [3], described the fatty acid requirements (n-3/n-6) in zebrafish larvae using larval growth as a proxy, and concluded that a diet with a low n-3/n-6 ratio maximizes growth. This finding is coincident with the requirement of typical warm water species, namely, a higher demand for n-6 PUFA, for example, tilapia.

While this study is based mainly on the use of the RNA/DNA ratio as a proxy for nutritional condition, there are other methods to validate results that could be used in future studies, such as the expression of genes related to growth, which can provide new tools for analyzing growth in fish [52]. The levels of gene expression related to myogenesis and ATP concentrations in rainbow trout are drastically reduced in response to starvation [50]. The analysis of microarrays also shows that starvation in rainbow trout also decreases the levels of gene expression related to lipid metabolism and immune response [53]. 

All the results obtained indicate that the marine basidiomycete strain *Rhodotorula* sp. CNYC4007 represents a potential feed and/or supplement for first feeding of zebrafish. Additionally, it can be incorporated in species like rotifers and *Artemia* sp. nauplii to be bioencapsulated to potentially improve the nutritional state of larvae. All trials carried out in our study considered a comparison between *Rhodotorula* sp. and commercial sources of fatty acid (formulated diets or emulsions) at different larval stages. In each case, zebrafish larvae fed with *Rhodotorula* sp. showed at least equal growth and nutritional condition than the larval treatment with the commercial alternative. These results are similar in magnitude to those reported in species with differences in fatty acid metabolism such as *Salmo salar* parr and *Sparus aurata* larvae, where fish oil replacement with alternative sources of DHA (*Schistochytrium* sp. and *Crypthecodinium cohnii*) was suggested [25,31]. These results are relevant, as in other species, nutritional studies on zebrafish have determined positive or negative influences of some food compounds [34] and correspond to the first step in the screening process for novel ingredients or additives with a potential use in aquaculture [35].

Previous studies on *Rhodotorula* yeast have proposed it as a source of carotenoids [54], bioactive substances [55] and lipids, but just for aliphatic18-carbon atom fatty acid chains [56]. The *Rhodotorula* sp. strain CNYC4007 is the only *Rhodotorula* species that has been reported as a DHA and EPA producer and to the best of our knowledge this is the first work in which a *Rhodotorula* yeast has been used as feed. Finally, this study seeks to contribute in the search for a sustainable source of PUFAs for feeding fish [57] to avoid the use of fish oil from over-exploited marine resources.

## 4. Materials and Methods

### 4.1. Obtaining and Producing the Strain Rhodotorula *sp.* CNYC4007

The strain used in this study has a 97% identity for the gene 18S ribosomal RNA (rRNA) of *Rhodotorula* sp. [27]. It was extracted from Caleta Maule, Biobío Region, Chile, and is stored in the National Collection of Yeast Cultures in the UK with the code CNYC4007. Two preparations were obtained from the strain and used in the experiments: lyophilized flour and microencapsulated, which were prepared with atomization drying using two stabilizers (maltodextrin and capsule), following the protocols established by Pino et al. [58]. Formulation and proximate composition of the strain *Rhodotorula* sp. and commercial diets C1 (Mikrovit Hi-Protein, Silesia, Poland) y C2-DHA (Algamac, Poland) used in experiments are detailed in Table 3.

### 4.2. Obtaining and Maintaining Danio rerio Larvae

A breeding stock of *D. rerio* wild type strain was maintained at 27 ± 1 °C with a 12:12 h light:dark photoperiod and constant filtration. Males and females of the same age were kept together in a glass aquarium with a density not exceeding four to five fish/L. The fish were fed twice daily with *Artemia* sp. nauplii (Utah strain; Aquafauna Bio-Marine Inc., Hawthorne, CA, USA) and TetraMin tropical flakes. To obtain the larvae needed for the experiments, spawning was induced at three-day intervals by placing plastic receptacles in the culture systems. The fertilized eggs were removed from the receptacles and washed twice, first with a mixture of 1× reconstituted saline solution (E3 medium: NaCl_2_ 19 mM; KCl 9 mM; CaCl_2_ 16 mM; MgSO_4_ 17 mM; Merck & Co., Inc., Kenilworth, NJ, USA) and methylene blue 0.01% (*w/v*) and then with 1× E3 medium. The healthy embryos were incubated in a temperature controlled camera (27 °C) until hatching occurred. 

In preliminary observations on early development of zebra fish larvae we assessed the exact moment when the larvae had the adequate mouth size to begin the feeding with rotifers and *Artemia* sp. nauplii. At six days post-fertilization the larvae had completed yolk absorption and were used in first-feeding experiments (larvae at six days post-yolk absorption) or continued maturing until reaching the phase required for the subsequent experiments, when the larvae were 12 days old (larvae at 12 days post-yolk absorption). Plastic wells (700 mL) were used as rearing chambers, and installed in the aquariums. To ensure optimal water quality, the wells were equipped with mesh walls to allow for water circulation. The remains of feed and fecal matter were regularly removed from the bottom of the wells. Wells of different sizes were used for the feeding experiments according to larval size. The wells were placed in a 50-L tank at 26 °C, with constant oxygenation and a light/darkness ratio 14:10. The water was partially changed every two days to maintain optimal quality (total ammonia nitrogen (TAN) = 0.2 mg L^−1^; conductivity = 201.9 μS cm^−1^, pH = 7.5, dissolved oxygen = 7.32 mg L^−1^).

### 4.3. Validation of the RNA/DNA Method for Danio rerio Larvae

An assay was conducted with first-feeding larvae (one day post-yolk absorption), when the larvae were 6 days old, to validate the RNA/DNA method to compare larvae fed with a standard commercial feed (C1; Mikrovit Hi-Protein Table 3) and larvae in starvation. Three larvae from each treatment (group) were randomly selected every day to determine RNA and DNA concentrations. The experiment lasted six days, when larvae were 11 days old, given that by this day there was 100% mortality of the larvae in starvation, because of which the statistical analysis was applied only up to the tenth day.

### 4.4. Estimating Larval Growth

Individual larvae were removed from the wells for analysis in all of the experiments. Cold anesthesia was used for observation and measurement. Larvae were placed individually in a petri dish over a gel pack bar frozen at −20 °C. Observations and measurements were made under a stereoscopic magnifying glass equipped with a digital camera (Canon EOS REBEL T3, Canon U.S.A., Inc., Huntington, NY, USA). Photographs of the larvae were analyzed using Image Pro-Plus 6.0 software, Media Cybernetics, Inc., Rockville, MD, USA. Magnifications of 10× or 12× was used to determine the total length depending on the size of the larvae. The distance from the mouth to the posterior point of the notochord was measured for pre-flexion larvae, while the distance from the mouth to the hypural plate (standard length) was used for more developed larvae. After measurement, the larvae were immediately placed in Eppendorf tubes and stored at −80 °C.

### 4.5. Experiments with First-Feeding Age Larvae

#### 4.5.1. Feeding with *Rhodotorula* sp. CNYC4007 Lyophilized in Flour and in Microcapsules

Thirty first-feeding age larvae (one day post-yolk absorption), when the larvae were six days old, they were placed in 700-mL wells. The experiment involved three treatments: (1) larvae fed with standard commercial feed (control group; C1); (2) larvae fed with flour lyophilized of the strain *Rhodotorula* sp. CNYC4007; and (3) larvae fed with flour microencapsulated of *Rhodotorula* sp. CNYC4007 (Table 1). A preliminary experiment established a biomass of 30 mg (for 30 larvae) as an adequate daily ration of commercial feed and the lyophilized flour and 150 mg for microencapsulate. The experiment lasted nine days during which unconsumed feed was removed daily. Every second day three larvae per replicate were removed, measured (detail point 4) and stored at −80 °C individually for subsequent molecular analysis (detail point 7).

#### 4.5.2. Larval Feeding with Bioencapsulation in *Brachionus* sp.

Rotifers used in this experiment were enriched following the protocol of Estudillo del Castillo et al. [16]. The assay involved three treatments with respective replicates: (1) larvae fed with non-enriched rotifers (control group; C1); (2) larvae fed with rotifers enriched with a commercial feed high in DHA (C2-DHA); and (3) larvae fed with rotifers enriched with flour lyophilized of *Rhodotorula* sp. CNYC4007. Each replicate involved 20 larvae in a 300-mL well with 0.2-mm mesh walls that allowed for the flow of water while preventing rotifers from escaping. On a daily basis, 1.000 enriched rotifers and 1.000 without enrichment were applied according to the treatment, following the individual ration. Six randomly selected larvae (three per replicate) were removed from each treatment, then observed, measured (see point 4) and stored individually at −80 °C for subsequent molecular analysis (see point 7).

### 4.6. Experiments with Larvae at 12 Days Post-Yolk Absorption

To carry out this experiment *Artemia* sp. (Utah strain; Aquafauna Bio-Marine Inc.) nauplii were enriched with *Rhodotorula* sp. CNYC4007 in the form of lyophilized flour. The time and conditions of the enrichment were defined by modifying the protocols from Silva [48]. Six hundred *Artemia* sp. nauplii (8 h post-hatching) were placed in 15-mL tubes containing 5 mL of seawater. The tubes were incubated with continuous light and strong aeration at 26 °C. Three mg of C2-DHA or 3 mg of flour lyophilized with CNYC4007 were added, respectively, to the two tubes. The tubes containing nauplii with and without enrichment were kept under the same conditions. The enrichment time was 16 h, after which the nauplii were sieved (88 µm), washed and re-suspended in 2 mL of distilled water to proceed with feeding the larvae.

The experimental design involved three treatments: (1) Larvae fed with *Artemia* sp. nauplii without enrichment (control group; C1); (2) Larvae fed with *Artemia* sp. nauplii enriched with C2-DHA (positive control group); and (3) Larvae fed with *Artemia* sp. nauplii enriched with flour lyophilized with *Rhodotorula* sp. CNYC4007. Thirty larvae were placed in 700-mL containers (3 replicates per treatment). The experiment lasted nine days. Every second day three larvae per replicate were removed, observed, measured and then stored individually at −80 °C for subsequent molecular analysis. In all the trials (flour, rotifers and *Artemia* sp. nauplii) at the moment of photographing each larvae the presence of food in the gut was visually verified and then stored for posterior molecular analysis.

### 4.7. Obtaining the RNA/DNA Ratio Used as a Proxy for the Condition of Danio rerio Larvae

The protocol from Clemmensen [59], with modifications, was used to extract larval nucleic acid. The larvae were treated with Tris-EDTA (Ethylenediaminetetraacetic acid) buffer (Tris-HCl 0.05 M; NaCl 0.1 M; EDTA 0.01 M at pH 8.0) and SDS (sodium dodecyl sulfate) at 20% *p/v*, after which proteinase K (20 mg/mL) was added and homogenized with a glass swab until a uniform solution was observed. The solution was then centrifuged for one minute and placed in a thermo-regulated bath (50 °C) for 10 min, after which the samples were transferred to −20 °C for 20 min. The procedure of heating and cooling the samples was repeated three times, following which the samples were centrifuged. Chloroform, phenol and isoamyl alcohol (24:25:1) was added and homogenized in an extraction chamber. After centrifugation for 15 min, the supernatant was transferred to a sterile Eppendorf tube. 

Nucleic acids were determined separately using the Quantifluor ONE dsDNA System (Promega Co., Fitchburg, WI, USA) for DNA and the Quantifluor RNA System (Promega Co., Fitchburg, WI, USA) for RNA. The samples were prepared according the suppliers protocols. Fluorescence was measured in a multi-detection microplate reader (BioTek, Winooski, VT, USA, model FL×800) under a blue optical channel with excitation of 492 nm and an emission of 540 nm.

### 4.8. Analysis of Fatty Acid Content in Artemia *sp.* nauplii

Polyunsaturated fatty acids were extracted from *Artemia* sp. nauplii by saponification reaction and quantified by HPLC following the methodology described by Li et al. [60]. To do this, 100 mg of *Artemia* sp. nauplii without enrichment, enriched with C2-DHA and flour lyophilized from the strain of *Rhodotorula* sp. CNYC4007 was used, each in triplicate. One mL of NaOH at 0.5 M in 96% of ethanol was added to the samples and homogenized with an Ultra Turrax for one minute. Samples were centrifuged at 7000 rpm for 5 min to eliminate solid residues after cell rupture. One mL of HCl at 0.6 N and 3 mL of ethyl acetate (LiChrosolv^®^, Rochester, NY, USA) was added to the supernatant and agitated with vortex for one minute and incubated for 30 min at room temperature. The treated samples were dried by a current of nitrogen (N_2(*g*)_) to eliminate the organic solvent, lyophilized to eliminate the remains of water and stored at −20 °C until subsequent chromatographic analysis. Polyunsaturated fatty acids were quantified with a HPLC VWR™ HITACHI (VWR International Ltd., Lutterworth, UK) with an organizer (model L-2000), an ultraviolet (UV) detector (model L-2400), gradient editing pump (model L-2100/2130) and a 15-cm-by-4.6-mm LC-18 column (Supelco^®^, Sigma-Aldrich, Inc., Darmstadt, Germany). The mobile phase consisted of a flow of 1 mL per min^−1^ of gradient A (25% acetonitrile), to 50% of gradient B (100% acetonitrile), with a flow of 2 mL per min^−1^ for the first 15 min and then 1 mL per min^−1^ for another 15 min. DHA and EPA were identified through the construction of their respective calibration curves using HPLC grade standards (Sigma-Aldrich^®^, Darmstadt, Germany) with an injection volume of 10 µL [27].

### 4.9. Statistical Analysis

The Statistica 10 program was used to analyze the data obtained. In the first step the homogeneity of variance (Bartlett, London, UK) and normality of data (Kolmogorov-Smirnov test) was analyzed. A two-way ANOVA and multiple Tukey comparison test was applied for total larval length, DNA and RNA concentrations and the RNA/DNA ratio. The treatments were compared on the final day of the assay. The Kruskal-Wallis test was applied to data that did not present homogeneity of variance or normality. Mean was considered significant when probability was less than 0.05 (*p* < 0.05).

## Figures and Tables

**Table 1:** Total larval length (average ± SD) for assay with six-day-old larvae of *Danio rerio* after post-yolk absorption. Larvae were fed with bioencapsulated *Brachionus* sp. rotifers and *Artemia* sp. nauplii. The daily average is based on six larvae per treatment. C1: without enrichment; C2-DHA: feed rich in docosahexaenoic acid (DHA) treatment; CNYC4007: *Rhodotorula* sp. CNYC4007 treatment.

Larvae Age (Days)	Total Length Larvae (mm)					
	*Brachionus* sp.			*Artemia* sp. nauplii		
	C1	C2-DHA	CNYC4007	C1	C2-DHA	CNYC4007
6	5.0 ± 0.2	5.0 ± 0.2	5.0 ± 0.2	n/a	n/a	n/a
8	5.2 ± 0.3	5.5 ± 0.4	5.1 ± 0.2	n/a	n/a	n/a
10	5.6 ± 0.1	5.6 ± 0.3	5.6 ± 0.3	n/a	n/a	n/a
12	5.6 ± 0.1	5.7 ± 0.2	5.8 ± 0.2	5.6 ± 0.3	5.6 ± 0.3	5.6 ± 0.3
14	n/a	n/a	n/a	5.6 ± 0.3	5.7 ± 0.3	5.8 ± 0.4
16	n/a	n/a	n/a	5.6 ± 0.2	6.0 ± 0.3	6.0 ± 0.4
18	n/a	n/a	n/a	6.6 ± 0.1	6.9 ± 0.3	7.0 ± 0.3
20	n/a	n/a	n/a	9.5 ± 0.4	10.2 ± 0.3	10.1 ± 0.7
22	n/a	n/a	n/a	9.9 ± 0.8	10.6 ± 0.3	10.6 ± 0.5

**Table 2:** RNA/DNA ratio for assay with six-day-old larvae of *Danio rerio* after post-yolk absorption. Larvae were fed with bioencapsulated *Brachionus* sp. rotifers and *Artemia* sp. nauplii. The daily average is based on six larvae per treatment. C1: without enrichment; C2-DHA: feed rich in DHA treatment; CNYC4007: *Rhodotorula* sp. CNYC4007 treatment.

Larvae Age (Days)	RNA/DNA Ratio					
	*Brachionus* sp.			*Artemia* sp. nauplii		
	C1	C2-DHA	CNYC4007	C1	C2-DHA	CNYC4007
6	1.1 ± 0.2	1.1 ± 0.2	1.1 ± 0.2	n/a	n/a	n/a
8	1.3 ± 0.2	1.1 ± 0.3	1.4 ± 0.3	n/a	n/a	n/a
10	1.2 ± 0.2	1.5 ± 0.1	1.5 ± 0.3	n/a	n/a	n/a
12	**1.0 ± 0.2**	**1.1 ± 0.1**	1.4 ± 0.2 *	0.8 ± 0.2	0.8 ± 0.2	0.8 ± 0.2
14	n/a	n/a	n/a	1.1 ± 0.1	0.9 ± 0.2	1.1 ± 0.3
16	n/a	n/a	n/a	1.0 ± 0.1	1.1 ± 0.2	1.1 ± 0.3
18	n/a	n/a	n/a	1.0 ± 0.2	1.1 ± 0.1	1.2 ± 0.2
20	n/a	n/a	n/a	0.8 ± 0.1	1.1 ± 0.1	1.1 ± 0.1
22	n/a	n/a	n/a	0.8 ± 0.1 *	**1.1 ± 0.1**	**1.0 ± 0.1**

* = *p*-value < 0.05; n/a = not available.

**Table 3:** Proximate composition of the diets used as a control (C1), positive control (C2-DHA) and the strain *Rhodotorula* sp. CNYC4007.

Components (%)	C1	C2-DHA	CNYC4007 Microencapsulated	CNYC4007 Meal
Protein	49	17.6	n/a	n/a
Carbohydrate	n/a	15.9	92.5	36.0
Fat	8.5	56.2	0.3	7.6
Nitrogen compounds	n/a	n/a	1.4	6.1
Fiber	3	n/a	1.2	1.7
Moisture	6	2.1	3.7	83.1
Ash	n/a	8.2	0.5	7.6
Calories (kcal)	n/a	640	378.2	169.8

n/a = not available.
